# Clustering the annotation space of proteins

**DOI:** 10.1186/1471-2105-6-24

**Published:** 2005-02-09

**Authors:** Victor Kunin, Christos A Ouzounis

**Affiliations:** 1 Computational Genomics Group, EMBL-EBI, Cambridge, CB10 1SO, UK

## Abstract

**Background:**

Current protein clustering methods rely on either sequence or functional similarities between proteins, thereby limiting inferences to one of these areas.

**Results:**

Here we report a new approach, named CLAN, which clusters proteins according to both annotation and sequence similarity. This approach is extremely fast, clustering the complete SwissProt database within minutes. It is also accurate, recovering consistent protein families agreeing on average in more than 97% with sequence-based protein families from Pfam. Discrepancies between sequence- and annotation-based clusters were scrutinized and the reasons reported. We demonstrate examples for each of these cases, and thoroughly discuss an example of a propagated error in SwissProt: a vacuolar ATPase subunit M9.2 erroneously annotated as vacuolar ATP synthase subunit H. CLAN algorithm is available from the authors and the CLAN database is accessible at

**Conclusions:**

CLAN creates refined function-and-sequence specific protein families that can be used for identification and annotation of unknown family members. It also allows easy identification of erroneous annotations by spotting inconsistencies between similarities on annotation and sequence levels.

## Background

To achieve high quality of annotation, curators are using direct evidence from additional experiments or infer functional roles by sequence similarity to experimentally characterized genes or proteins. However, erroneous annotations generated at early stages may propagate to new homologous sequences, ultimately leading to erroneous annotation of entire families. The number of database errors is known to grow with time, as the number of entries and errors tend to accumulate. A recent analysis suggested that as databases grow annotation errors may propagate at an exponential rate [1].

One of the most accurate and consistently annotated databases is SwissProt [2] – a manually curated protein sequence database which strives to provide a high level of annotations such as the description of the function of a protein, its domain structure, post-translational modifications, variants, etc. All entries in SwissProt are annotated by experts, thus reducing the amount of errors expected from fully automatic methods.

This impressive collection of textual information provides excellent opportunities for natural language processing in computational biology. Text mining of the free text in biomedical literature is well established (see [3] for a recent review). These methods use standard techniques such as the TF-IDF method, which considers background frequency of terms and the frequency of terms in the documents of interest. However, protein annotations are conceptually different from the free text of biomedical articles and abstracts upon which these methods are applied.

While articles normally contain thousands of words and abstracts contain a few hundred, annotations are limited to a few key terms, averaging 7 and rarely exceeding 20 words. Research articles are always unique, whereas orthologous proteins often have identical annotations. On the other hand, differences in annotations may or may not signal difference in function, depending on the context. For example, the addition of the word "precursor" to the annotation does not imply functional variation. Also, multiple occurrence of a term in an article or an abstract signals its higher relevance to the subject. In contrast, presence of a term in a protein annotation normally signals presence of a function, and its repetition is meaningless. All these differences require the development of a different method for clustering protein annotations.

This work describes a method called CLAN specially developed to assess the consistency of annotations. CLAN allows the rapid comparison of protein annotations, finding all proteins that are considered to have the same (or a closely related) function, according to the functional descriptions of the corresponding database entries. To group pairwise hits between these proteins, clustering of connected components was used, and sequence similarity within the clusters was considered. Using CLAN, an exemplar error was identified in the annotation of the M9.2 (M9.7) subunit of a vacuolar H+ ATPase (V-ATPase).

## Results

### General statistics

**Figure 1:**
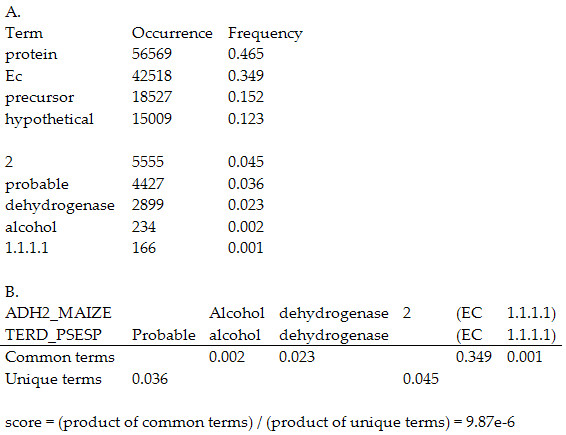
**Score calculation. **An example of calculation of the score by CLAN. A. In the pre-processing stage, a dictionary is constructed with occurrences of terms in the SwissProt. Multiple occurrence of a term in a single entry is counted once. Frequency is calculated by dividing the term occurrence to the number of entries in the database. Numbers are rounded to the third decimal digit. B. An example of calculation of score for an alignment of two actual annotations.

We used CLAN [see Methods] to cluster SwissProt entries based on annotation strings. We experimented with score cut-offs [see Figure 1 and Methods for derivation of scores] between 10^-2 ^and 10^-6^. Depending on the threshold, the output contained between 2,070,221 (for *e *≤ 10^-6^) and 3,980,763 (*e *≤ 10^-2^) pairwise annotation similarities. The output of CLAN was subjected to clustering of connected components, resulting in 43,416 (*e *≤ 10^-2^) up to 47,344 (*e *≤ 10^-4^) clusters, including singletons. When a lower score threshold was used, the number of clusters in the output decreased, as more proteins were excluded from any clusters (see below).

A sample of few hundred of the annotation clusters was examined manually to assess whether they contained proteins annotated as functionally unrelated. At the low score cut-offs, we failed to find any such examples, thus suggesting a high degree of specificity. Also, hypothetical proteins were correctly not grouped together, which reflects their singularity in the protein function space. However, at these cut-off values, proteins annotated with a single word (i.e. Plastocyanin) failed to pass the threshold of meaningful annotation. This effect is due to the fact that no single term has a sufficiently low frequency to provide an score below the threshold. In total, 29,845 entries were not even defined as singletons (these include both characterized and hypothetical proteins) as their annotation strings are non-descriptive, resulting in high scores above the threshold.

On the other hand, at the highest score threshold the method succeeds in clustering together many single-word annotations. However, this increase in sensitivity is counter-balanced by a decrease in specificity. At the score of 10^-2^, the biggest cluster contains proteins annotated as "Hypothetical *X *kDa protein in *Y *intergenic region", where *X *is the calculated protein molecular weight and *Y *is the genomic location. These proteins are most probably unrelated, perform different functions or may not be expressed at all, and thus should not be clustered together. In fact, this cluster is eliminated at score cut-off of 10^-3^.

### Comparison to Pfam database

To estimate how well annotation clusters correspond to the sequence-based protein families, we compared the CLAN clusters (*e *≤ 10^-2^) to the Pfam-A database [4]. This database is a curated semi-automatic collection of protein domains, defined by experts as sharing sequence (and usually functional) similarity. To perform a meaningful comparison, we used only CLAN clusters containing more than 3 members. We found that on average 91% of CLAN cluster members belong to a single Pfam family.

However, the reverse relationship does not hold: only 51% of Pfam entries correspond to a single CLAN cluster, on average. This suggests that annotation-based clusters are about twice as small and contain only parts of sequence-based families. This is partially due to inconsistent annotation of protein families discussed below. On the other hand, it also reflects multifunctionality and diversification of some protein families. For example, a single Malate and Lactate dehydrogenase (MDH/LDH) family from Pfam is divided by CLAN to distinct annotation families, thus providing a finer distinction between family members that can be used for more accurate annotation of functional specificity [5].

As many of the CLAN clusters which correspond to several Pfam families contain multi-subunit complexes or multidomain proteins, a rigorous domain detection procedure is expected to drastically improve this result. For the results of the combination of CLAN annotation clusters with sequence-based clustering, see the section describing the CLAN database.

Though SwissProt annotation is not independent from Pfam assignments and often depend on them, a few clusters were found that did not correspond to Pfam families. Thus, CLAN system might facilitate identification and rapid incorporation of new families to curated protein family databases such as Pfam.

### Analysis of individual clusters

We aimed to determine whether similarity of the annotation corresponds to the sequence similarity of the proteins. For this, we compared protein sequences within annotation clusters using BlastP program [6]. Overall, we found very consistent sequence similarity relationships within annotation clusters. The proteins participating in the annotation clusters usually formed tight protein families, with all (or most) proteins sharing detectable sequence similarity to the other cluster members. This pattern is expected, since protein annotation is usually based upon sequence similarity. We considered this pattern to be typical and examined any deviations from it.

Analysis of sequence similarity within annotation-based families identified the following exceptions to the consistent similarity patterns between all family members.

1. Fragments of proteins. The fragments often fail to produce significant Blast scores, thus appearing as singletons on the similarity graph.

2. Taxa – specific forms of enzymes that form separate protein families, such as Bacterial and Archaeal adenylate kinases, forming distinctive sequence-based families.

3. Non-orthologous gene displacements, such as malate dehydrogenases.

4. Protein complexes. Annotation – derived families often included multiple chains/subunits of protein complexes, such as Glutamyl-tRNA(Gln) amidotransferase subunits A, C, D and E.

5. False positives of CLAN. The only case found so far involves the 'hypothetical protein' cluster, appearing at the permissive score cut-off 0.01 (see previous section).

6. SwissProt annotation errors.

### Fragments

SwissProt contains 7,232 entries annotated as "fragment". These are normally short fragments of larger protein molecules. An extreme example is LUXE_VIBFI (3 residues only), a fragment of Long-chain-fatty-acid-luciferin-component ligase. The complete sequence from a related *Vibrio *species documented in SwissProt (LUXE_VIBHA) contains 378 residues. There are at least 71 entries annotated as "fragment", whose sequences are less then 10 residues long. As their sequence often contains a subset from full-length close homologs, the information from these fragments is often redundant. Also, these short fragments fail to produce significant scores in any sequence similarity search procedure, and thus are usually irrelevant for sequence search engines. Moreover, fragments often create noise in sequence alignments and HMM analyses creating pseudo-conserved domains, and pseudo-gaps. In summary, we question the value of the presence of short protein fragments in a manually curated database, aiming for maximum precision rather then maximum coverage.

### Taxa-specific forms of enzymes

In some taxa, proteins performing identical functions might diverge to a point where they are defined as separate sequence-based families, sharing marginal similarity. One such example is the bacterial and archaeal adenylate kinases. Though similar at the 3-dimensional level [7], the sequences of members of these families diverged beyond recognition by BlastP, and are thus considered as separate families.

### Non-orthologous gene displacements

Enzymes belonging to different structural families may perform identical cellular function. In this case, annotation does not provide enough information about the type of the family a particular enzyme belongs to. One such case is represented by glucokinases, which belong to different families but perform identical function. For example, GNTK_BACSU and GNTK_ECOLI are both glucokinases and are annotated as such, were thus clustered together by CLAN but are unrelated to each other [8].

### Protein complexes

Subunits in protein complexes often have almost identical annotation. For example, Glutamyl-tRNA(Gln) amidotransferase subunits A, C, D, and E differ only in the chain identifier. The annotation of the subunits is somewhat misleading, as subunits A, B and C form one form of a rather ubiquitous complex [9], and subunits D and E form an alternative archaeal-specific complex [10]. Each of the chains forms a tight sequence-similarity based family, with no similarity detected between chains. Entries describing chains A, C, D and E appear in a single CLAN cluster. Interestingly, a few organisms have the entire complexes annotated in SwissProt (Table 1), although in many cases the documentation of these protein complexes is incomplete. The CLAN approach can help to identify the organisms where the chains remain to be identified, while the phylogenetic pattern of the complex may serve as an additional evidence for further annotation.

### Annotation errors

Finally, CLAN can serve as a powerful tool to identify annotation errors. Overall, we found SwissProt annotations very consistent and robust. Most of the annotation-based sets resulted in tight sequence-based similarity clusters. A few cases where no similarity was found between a protein and the rest of the cluster were normally due to the limits in sensitivity by BlastP, and the homology was clear when multiple alignments, profile- or HMM-based search methods were used. Nevertheless, there are cases where annotation is problematic. Below, we discuss in detail an example of inconsistency found in the annotation of a cluster containing vacuolar H-ATPase.

### False annotation example: Vacuolar ATP synthase subunit M9.2

When analyzing the output of CLAN with score threshold 10^-6^, we found an interesting example of a propagated error. The protein VAOH_CAEEL (Q20591) from *Caenorhabditis elegans *has been annotated as "Probable vacuolar ATP synthase subunit H (EC 3.6.3.14) (V-ATPase H subunit) (Vacuolar proton pump H subunit)". This protein is clustered by CLAN with other vacuolar ATP synthase subunits. However, BlastP alignment failed to produce evidence of similarity between this protein and other members of the cluster annotated as vacuolar ATP synthase subunit H. Also, this protein is much shorter then other members of the annotation cluster, comprising only of 96 residues, while other members of the cluster have more then 400 residues. In fact, *C. elegans *was reported to contain another two V-ATPase H subunits, with length of 451 and 470 residues that share substantial sequence similarity with other members of the family. Multiple alignments of the family suggested that VA0H_CAEEL is a distant protein not related to the rest of the family. The SwissProt entry does not contain a link to a published work. A PSI-Blast search against the NCBI NR database allowed to identify other members of the family. The family was first described in the literature as "M9.2 V-ATPase subunit" in bovine [11]. Interestingly, an appropriate sequence entry exists in SwissProt with an adequate reference (VA0H_BOVIN). However, this protein is named in SwissProt as "vacuolar ATP synthase subunit H", and the name V-ATPase M9.2 subunit is suggested as a synonym. Another SwissProt entry (VA0H_HUMAN) contains a human homolog, with identical annotation. Interestingly, the bovine and human proteins were unified into a separate annotation cluster at score 10^-6^, which was unified with other members of "vacuolar ATP synthase subunit" cluster at lower score cut-offs.

The M9.2 V-ATPase subunits were identified in mammals [11,12], insects [11,13] and *C. elegans *[11]. The alternative names included "M9.7" or subunit 'e' in insects [14,15] and ATP6H in dog [12]. A potential homolog was identified in the plant *Mesembryanthemum crystallinum *(gi 26986112) [16]. Using PSI-BLAST searches we have identified homologs in several other species, including *Arabidopsis thaliana*, *Anopheles gambiae *and a few paralogs in *Drosophila melanogaster*. The multiple alignment of these proteins is provided as supplementary data.

The M9.2 protein was suggested to be a homolog [11] of yeast Vma21p protein (VM21_YEAST). The Vma21p protein is required for assembly of the V-ATPase, but is not found in the mature complex [17]. The orthology of the two proteins was suggested based on the weak sequence identity, association with V-ATPase complex, and a similar hydrophobicity profile [11]. However, the doubts concerning orthology of the two proteins were already expressed [14]. While Vma21p is localized in endoplasmic reticulum and is not a part of the mature V-ATPase [17], M9.2 is a subunit of mature V-ATPase located in the vacuolar membrane [11]. In addition, the topology of the yeast protein seems to be inverse to that of the mammalian and insect proteins: glycosylation at the C-terminus of the *M. sexta *protein indicates that the C-terminus is exposed to the extracellular surface, whereas the C-terminus of Vma21p appears to be localized on the cytosolic side of the membrane [14]. To identify any similarity between these two protein families, we used sequence-versus-sequence (BlastP), sequence to profile (PSI-BLAST) [6], and profile-profile (LAMA) [18] search methods. Each of these methods failed to detect any significant similarity between the two families. Our sequence analysis suggests that the initial reports of such homology between these families were not substantial, and the reported marginal similarity between the two sequences was due to the extent of hydrophobic regions in the two protein families. In summary, while the exact function of the M9.2 subunit of V-ATPase is still not known, it has a turbulent history of misinterpretation. It was confused with V-ATPase H chain in SwissProt, it was misaligned with yeast Vmi21p protein, it has several names in different organisms of which the name 'subunit e' is most confusing, as there is already a 'subunit E' in the complex. The current study used (i) CLAN to detect the discrepancies for protein annotation in SwissProt and (ii) sensitive sequence searches in order to demonstrate the independent status of this protein family.

The error in the annotation of M9.2 subunit of V-ATPase was reported to the SwissProt developers, and is likely to be corrected at the time of publication of this work.

### False negative example

Another form of annotation error results in a false negative case for CLAN. It occurs when a protein clearly belongs to a certain family but is not annotated as such. This event can be detected when several CLAN families map to a single sequence similarity-based family. One example is SUCD_BACSU, annotated as " Succinyl-CoA synthetase alpha chain (EC 6.2.1.5) (SCS-alpha)". This protein belongs to the SUCD family of proteins, and should cluster with its other members. However, its annotation contains synonyms " (Vegetative protein 239) (VEG239)", which makes this protein virtually unique in annotation space. Another well-known example is the initial characterization of some general stress proteins in bacteria, later shown to be ribosomal proteins (e.g. S1, L25) [19] – the initial annotations have not been modified. This type of assignments are usually based on genetics experiments and do not reflect the precise biochemical function of these proteins. In fact, there are multiple examples of proteins having unique or phenotype-based identifiers in their names, whereas it is clear that these proteins belong to larger protein families of known biochemical function. In our opinion, these proteins are examples of conflicting annotation with other family members and their annotations should be amended.

## The clan database

Based on the results described above, we designed a database using the CLAN output with score threshold of 10^-2^. The CLAN database contains two levels of clustering. The first level contains clusters based on comparison of protein annotations, as described in the Methods section. To ensure completeness and reliability of the results, any protein annotated as 'fragment' was excluded from further processing. The second level in the database is generated by applying sequence-similarity-based grouping to each of the clusters obtained in the first stage. All members within annotation clusters are aligned using BlastP. The output of BlastP is subjected to various clustering procedures. In order to distinguish between the annotation-based clusters of the first database level, and the sequence-based sub-clusters of the second level, we call these 'clusters' and 'sub-clusters' accordingly. When referring to both these categories simultaneously, we use the notation (sub)clusters.

To estimate the contribution from the second level of clustering, we repeated the comparison to Pfam database, using sub-clusters instead of clusters. We found that on average 97% of CLAN sub-clusters members belong to a single Pfam family, (compared to the 91% for clusters). The gain in selectivity was accompanied by gain in sensitivity, and 66% of Pfam family members corresponded to a single sub-cluster (compared to 51% for clusters). Compared to annotation-based clustering alone, the dramatic increase in the sensitivity reflects the fact that in the second level of clustering, non-homologous sequences are removed and form separate sub-clusters.

The CLAN database has a web-based interactive interface, located at . To facilitate database navigation, we introduced (sub)cluster names, computed as a minimal consensus string for all (sub)cluster members. The MySQL-based storage of data allows fast searches based on sequence or cluster name, SwissProt accession or cluster identifier. The sequence similarity within clusters can be examined visually using the BioLayout software [20]. To facilitate the annotation of protein complexes, phylogenetic distribution of clusters between sub-clusters (such as Table 1) can be automatically generated upon the user's request. For all sub-clusters full-length multiple alignments are pre-computed with the ClustalW program [21], and the alignments can be viewed via an interactive Java interface.

## Discussion

We have presented CLAN, a rapid and powerful method to build consistent annotation-based families, and assessed the consistency of annotation in protein databases. We have also presented the CLAN database, as a collection of protein families grouped by function. We believe that these tools can be used to improve the quality of database curation and assist annotation of newly sequenced proteins.

The double-step clustering presented here has resulted in the creation of a reliable database of protein families, with include proteins similar on both annotation and sequence levels. One of the potential uses of this database is an accurate assignment of function to newly sequenced proteins by protein – to – profile alignments or HMMs. Our method has the potential to distinguish between subtle changes that might reflect diversification of protein function. For example, homologous Malate and Lactate dehydrogenases are found in different (sub)clusters, allowing their characterization as separate protein families. As most protein family databases group proteins by homology, as opposed to function, to the best of our knowledge, our approach is unique in its ability to build protein sequence families distinguishing between functions. In the future, other such comparisons could include other structural classifications such as CATH [22] or TRIBEs [23] and functional classifications such as EC or GO [24].

## Methods

We used SwissProt release of Feb 13, 2003 that contained 121,744 protein sequences. We aimed to find the similarity between the database entries solely based on the annotation and without any reference to sequence similarity. The annotation corpus considered here consisted of description line (protein name) and synonyms from the SwissProt database. We interchangeably use the term 'function' or 'annotation' to denote the contents of this corpus, implying the use of the corresponding annotation strings from the database.

### Definition of distance measure between annotation fields

To obtain a distance measure, we first calculated the frequency *φ *of each word as the number of database entries containing the word *N *divided by the total number of entries in the database *D *(Equation 1). The word frequencies served as weights, because the words describing specific functions (such as "plastocyanin") occur less frequently than non-specific words (such as "protein").


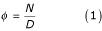


We aimed to devise a scoring scheme that would produce highly significant scores for proteins with related function, while being able to distinguish between proteins with different functions. The score for the common terms *p *between protein functions may be computed as the product of frequencies of *n *terms shared between the two annotations (Equation 2).


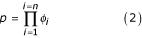


However, sharing only part of the total number of significant words is insufficient: proteins may use similar substrates for different reactions, or perform similar chemical reactions on different substrates. There may be three proteins with annotations AB, BC, and CD respectively, and clustering by similarity only could lead to clustering together AB and CD. For example, "lactate dehydrogenase" should be differentiated from "lactate permease" or "alcohol dehydrogenase".

To achieve this distinction, we computed the difference between the two annotations, as above, for the case of the similarity measure. The score for the unique terms *q *is the product of frequencies of *m *terms unique to any of the two annotations (Equation 3).


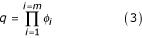


Finally, the distance *e *between the two annotations is defined as the fraction between the score for common terms *p *and the score for unique terms *q *for the two annotation strings (Equation 4). We refer to this scoring function as 'score' throughout the manuscript.


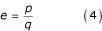


An example of calculation of the score is shown on Figure 1.

### Implementation

The procedure calculating pairwise distances between protein annotations described above was implemented in a perl program. Its sole input consists of SwissProt identifiers and annotation lines, and the output contains pairs of proteins with the score of annotation similarity lower than a given threshold. Compared to sequence-similarity clustering, this procedure is very rapid: an all-against-all comparison of SwissProt database containing 121,744 entries took about 26 minutes on a single processor of a Sun-Fire-480R server with 2 GB of RAM.

The result of the pairwise comparisons between protein annotations were then subjected to clustering of connected components. The protein sequences of the clusters obtained were subsequently analyzed with the BlastP program [6] and the results of similarities were clustered using GeneRage algorithm [25]. The results were visualized using the BioLayout software [20] (and Goldovsky, Cases *et al., *submitted). To evaluate the ability of CLAN to detect genuine protein families on the basis of annotation alone, the output (*e *≤ 10^-2^) was compared to the Pfam-A database release 9.0 [4]. For each CLAN cluster with more then 3 entries, we have identified the corresponding Pfam entry (if available), and counted the fraction of CLAN entries found in the same Pfam cluster. The reverse comparison was computed in a similar manner.

## Figures and Tables

**Table 1:** Phylogenetic distribution of Glutamyl-tRNA(Gln) amidotransferase subunits as represented in SwissProt. Note that the table is not completely populated. Evidence of presence of part of the complex can assist annotation for absent members.

	chain A	chain B	chain C	chain D	chain E
Aeropyrum pernix	GATA_AERPE	GATB_AERPE		GATD_AERPE	GATE_AERPE
Agrobacterium tumefaciens (strain C58 / ATCC 33970)		GATB_AGRT5			
Anabaena sp	GATA_ANASP	GATB_ANASP	GATC_ANASP		
Aquifex aeolicus	GATA_AQUAE	GATB_AQUAE	GATC_AQUAE		
Archaeoglobus fulgidus	GATA_ARCFU	GATB_ARCFU	GATC_ARCFU	GATD_ARCFU	GATE_ARCFU
Bacillus halodurans	GATA_BACHD	GATB_BACHD	GATC_BACHD		
Bacillus stearothermophilus	GATA_BACST	GATB_BACST	GATC_BACST		
Bacillus subtilis	GATA_BACSU	GATB_BACSU	GATC_BACSU		
Borrelia burgdorferi	GATA_BORBU	GATB_BORBU	GATC_BORBU		
Brucella melitensis		GATB_BRUME	GATC_BRUME		
Brucella suis		GATB_BRUME	GATC_BRUME		
Campylobacter jejuni	GATA_CAMJE	GATB_CAMJE	GATC_CAMJE		
Caulobacter crescentus		GATB_CAUCR	GATC_CAUCR		
Chlamydia muridarum	GATA_CHLMU	GATB_CHLMU	GATC_CHLMU		
Chlamydia pneumoniae	GATA_CHLPN	GATB_CHLPN	GATC_CHLPN		
Chlamydia trachomatis	GATA_CHLTR	GATB_CHLTR	GATC_CHLTR		
Chlorobium tepidum		GATB_CHLTE			
Clostridium acetobutylicum		GAB1_CLOAB GAB2_CLOAB	GAC1_CLOAB GAC2_CLOAB		
Corynebacterium glutamicum		GATB_CORGL			
Deinococcus radiodurans		GATB_DEIRA	GATC_DEIRA		
Fusobacterium nucleatum (subsp nucleatum)	GATA_FUSNN	GATB_FUSNN			
Halobacterium sp		GATB_HALN1	GATC_HALN1	GATD_HALN1	GATE_HALN1
Helicobacter pylori	GATA_HELPY	GATB_HELPY	GATC_HELPY		
Helicobacter pylori J99	GATA_HELPJ	GATB_HELPJ	GATC_HELPJ		
Lactococcus lactis (subsp lactis)	GATA_LACLA	GATB_LACLA	GATC_LACLA		
Listeria innocua	GATA_LISIN	GATB_LISIN	GATC_LISIN		
Listeria monocytogenes	GATA_LISMO	GATB_LISMO	GATC_LISMO		
Methanobacterium thermoautotrophicum	GATA_METTH	GATB_METTH		GATD_METTH	GATE_METTH
Methanococcus jannaschii	GATA_METJA	GATB_METJA	GATC_METJA	GATD_METJA	GATE_METJA
Methanopyrus kandleri		GATB_METKA		GATD_METKA	GATE_METKA
Methanosarcina acetivorans	GATA_METAC	GATB_METAC		GATD_METAC	GATE_METAC
Methanosarcina mazei	GATA_METMA	GATB_METMA		GATD_METMA	GATE_METMA
Moraxella catarrhalis	GATA_MORCA	GATB_MORCA	GATC_MORCA		
Mycobacterium leprae	GATA_MYCLE	GATB_MYCLE	GATC_MYCLE		
Mycobacterium tuberculosis	GATA_MYCTU	GATB_MYCTU	GATC_MYCTU		
Mycoplasma genitalium	GATA_MYCGE	GATB_MYCGE			
Mycoplasma pneumoniae	GATA_MYCPN	GATB_MYCPN			
Mycoplasma pulmonis		GATB_MYCPU			
Neisseria meningitidis (serogroup A)	GATA_NEIMA	GATB_NEIMA	GATC_NEIMA		
Neisseria meningitidis (serogroup B)	GATA_NEIMB	GATB_NEIMB	GATC_NEIMB		
Pseudomonas aeruginosa	GATA_PSEAE	GATB_PSEAE	GATC_PSEAE		
Pyrobaculum aerophilum				GATD_PYRAE	GATE_PYRAE
Pyrococcus abyssi				GATD_PYRAB	GATE_PYRAB
Pyrococcus furiosus				GATD_PYRFU	GATE_PYRFU
Pyrococcus horikoshii				GATD_PYRHO	GATE_PYRHO
Ralstonia solanacearum		GATB_RALSO	GATC_RALSO		
Rhizobium loti		GATB_RHILO	GATC_RHILO		
Rhizobium meliloti		GATB_RHIME	GATC_RHIME		
Rickettsia conorii	GATA_RICCN	GATB_RICCN	GATC_RICCN		
Rickettsia prowazekii	GATA_RICPR	GATB_RICPR	GATC_RICPR		
Saccharomyces cerevisiae	GATH_YEAST				
Schizosaccharomyces pombe	GATH_SCHPO				
Staphylococcus aureus	GATA_STAAU	GATB_STAAU	GATC_STAAM		
Staphylococcus sp		GATB_STASP			
Streptococcus pneumoniae	GATA_STRPN	GATB_STRPN	GATC_STRPN		
Streptococcus pyogenes	GATA_STRPY	GATB_STRPY	GATC_STRPY		
Streptomyces coelicolor	GATA_STRCO	GATB_STRCO	GATC_STRCO		
Sulfolobus solfataricus	GATA_SULSO	GATB_SULSO	GATC_SULSO	GATD_SULSO	GATE_SULSO
Sulfolobus tokodaii	GATA_SULTO	GATB_SULTO	GATC_SULTO	GATD_SULTO	GATE_SULTO
Synechocystis sp	GATA_SYNY3	GATB_SYNY3	GATC_SYNY3		
Thermoanaerobacter tengcongensis	GATA_THETN	GATB_THETN			
Thermoplasma acidophilum				GATD_THEAC	GATE_THEAC
Thermoplasma volcanium				GATD_THEVO	GATE_THEVO
Thermotoga maritima	GATA_THEMA	GATB_THEMA	GATC_THEMA		
Thermus thermophilus	GATA_THETH	GATB_THETH	GATC_THETH		
Treponema pallidum	GATA_TREPA	GATB_TREPA	GATC_TREPA		
Ureaplasma parvum		GATB_UREPA			
